# Osteopontin and Clinical Outcomes in Hemodialysis Patients

**DOI:** 10.3390/biomedicines12112605

**Published:** 2024-11-14

**Authors:** Claudia Torino, Federico Carbone, Patrizia Pizzini, Sabrina Mezzatesta, Graziella D’Arrigo, Mercedes Gori, Luca Liberale, Margherita Moriero, Cristina Michelauz, Federica Frè, Simone Isoppo, Aurora Gavoci, Federica La Rosa, Alessandro Scuricini, Amedeo Tirandi, Davide Ramoni, Francesca Mallamaci, Giovanni Tripepi, Fabrizio Montecucco, Carmine Zoccali

**Affiliations:** 1 Clinical Epidemiology of Renal Disease and Hypertension Unit, Reggio Cal CNR Unit of the Pisa CNR Institute of Clinical Physiology, 89124 Reggio Calabria, Italy; claudia.torino@cnr.it (C.T.); patrizia.pizzini@cnr.it (P.P.); sabrinamezzatesta@cnr.it (S.M.); graziella.darrigo@cnr.it (G.D.); francesca.mallamaci@libero.it (F.M.); giovanniluigi.tripepi@cnr.it (G.T.); 2 First Clinic of Internal Medicine, Department of Internal Medicine, University of Genoa, 6 viale Benedetto XV, 16132 Genoa, Italy; federico.carbone@unige.it (F.C.); luca.liberale@unige.it (L.L.); 4307262@studenti.unige.it (M.M.); cristinamichelauz@gmail.com (C.M.); federica.fre96@gmail.com (F.F.); simone.isoppo.94@gmail.com (S.I.); aurora.gavoci@gmail.com (A.G.); fedef.laros@gmail.com (F.L.R.); alessandro.scuricini@gmail.com (A.S.); amedeo.tirandi@edu.unige.it (A.T.); davide.ramoni@edu.unige.it (D.R.); fabrizio.montecucco@unige.it (F.M.); 3 IRCCS Ospedale Policlinico San Martino, Genoa-Italian Cardiovascular Network, 10 Largo Rosanna Benzi, 16132 Genoa, Italy; 4 CNR—Institute of Clinical Physiology, 00186 Rome, Italy; mercedes.gori@cnr.it; 5 Nephrology, Hypertension and Renal Transplantation Unit, Grande Ospedale Metropolitano, 89124 Reggio Calabria, Italy; 6 Renal Research Institute, New York, NY 10065, USA; 7 IPNET, c/o Nefrologia del Grande Ospedale Metropolitano, 89124 Reggio Calabria, Italy

**Keywords:** OPN, hemodialysis, clinical outcomes, cardiovascular, mineral metabolism, biomarker

## Abstract

Background/Objectives: Chronic kidney disease (CKD) and end-stage kidney disease (ESKD) are significant public health issues, with cardiovascular morbidity and mortality being the leading causes of death in hemodialysis patients. Osteopontin (OPN), a multifunctional glycoprotein, has emerged as a potential biomarker for vascular disease in CKD due to its role in inflammation, tissue remodeling, and calcification. Methods: This cohort study included 1124 hemodialysis patients from the PROGREDIRE study, a registry involving 35 dialysis units in Southern Italy. Serum osteopontin levels were measured using enzyme-linked immunosorbent assay (ELISA). The primary endpoints were all-cause and cardiovascular mortality. Multivariate Cox regression analyses were performed to assess the association between osteopontin levels and mortality, adjusting for traditional risk factors, biomarkers of inflammation, nutritional status, and ESKD-related factors. Results: During a mean follow-up of 2.8 years, 478 patients died, 271 from cardiovascular causes. Independent correlates of osteopontin included alkaline phosphatase and parathyroid hormone. Elevated osteopontin levels were significantly associated with increased all-cause mortality (HR 1.19, 95% CI 1.09–1.31, *p* < 0.001) and cardiovascular mortality (HR 1.22, 95% CI 1.08–1.38, *p* = 0.001) after adjusting for confounders. Conclusions: Elevated osteopontin levels are associated with increased all-cause and cardiovascular mortality in hemodialysis patients. These findings implicate osteopontin in the high risk for death and cardiovascular disease in the hemodialysis population. Intervention studies are needed to definitively test this hypothesis.

## 1. Introduction

Chronic kidney disease (CKD) is a significant public health issue that affects millions of people worldwide [1]. Its most severe form, end-stage kidney disease (ESKD) often requires renal replacement therapies, such as three-weekly hemodialysis, to sustain life. Unfortunately, patients undergoing hemodialysis are at a particularly high risk of cardiovascular complications, which are the leading causes of death in this population. This increased risk is partly due to factors like vascular calcification and inflammation, which are exacerbated in the CKD and hemodialysis populations [2]. Osteopontin (OPN) is a 44–75 KD multifunctional phosphoprotein secreted by several cell types and present in the extracellular matrix of mineralized tissues and extracellular fluids, particularly at sites of inflammation [3,4]. OPN is expressed in various tissues, and it has recently emerged as a potential biomarker for increased cardiovascular risk in individuals with CKD, particularly those undergoing dialysis [5,6]. OPN is encoded by the secreted phosphoprotein 1 (*SPP1*) gene, located as a tandem array on the long arm of chromosome 4 [7]. This gene produces various splicing variants of mRNA, including full-length OPN and spliceosomal OPN (which is missing exons 4 or 5) [8,9]. The full-length OPN is cleaved by thrombin to form thrombin-cleaved OPN [10]. Both native OPN and cleaved OPN can interact with integrins, playing a crucial role in regulating inflammation, biomineralization, bone remodeling, immune functions, chemotaxis, and cell apoptosis [10,11]. OPN plays a critical role in pathways related to inflammation, tissue remodeling, and calcification—processes that are intimately linked to cardiovascular pathology [12].

Clinical studies have demonstrated that OPN plays a significant role in bone strength and remodeling, indicating that serum OPN levels are positively correlated with the severity of osteoporosis (OP). This suggests that OPN could be targeted as a biomarker for the early diagnosis of postmenopausal osteoporosis [13,14]. Additionally, OPN is associated with bone turnover and bone mineral density (BMD), influencing both morphological formation and reconstruction of bone [15,16,17]. Elevated serum OPN levels may lead to lower BMD and increased OP in postmenopausal women [16,17]. Furthermore, high levels of OPN are linked to osteoporotic fractures in this population, particularly in the lumbar spine [14].

While OPN has been studied in the general population as a biomarker for a variety of diseases, including cancer, autoimmune disorders [18], vascular calcification, and atherosclerosis [19], its role in cardiovascular disease in hemodialysis patients remains an emerging area of research [19]. Vascular stiffness and calcification are particularly common in patients on hemodialysis and contribute significantly to their elevated cardiovascular risk [20]. OPN is known to regulate mineral metabolism and vascular calcification, processes that are often disrupted in individuals with CKD [12]. Furthermore, the chronic inflammatory state characteristic of ESKD may be exacerbated by OPN, which can further elevate cardiovascular (CV) risk in these patients [18].

Brief reports from earlier studies have suggested a link between elevated OPN levels and arterial disease or vascular calcification in hemodialysis patients, but these studies have typically been limited in scale. For instance, a study involving 36 middle-aged hemodialysis patients indicated a possible association between OPN and vascular calcification [5]. However, large-scale studies examining the relationship between OPN levels and mortality outcomes in hemodialysis patients are lacking. In this cohort study, we investigated the association between circulating OPN levels and both all-cause and cardiovascular mortality in a sample of 1124 ESKD patients on chronic hemodialysis, finding a significant and direct association of this biomarker with the considered outcomes. The findings of this study could provide critical insights into the potential utility of OPN as a biomarker for predicting mortality risk and guiding clinical management in this vulnerable population.

## 2. Materials and Methods

The study protocol was approved by the ethical committee “Comitato Etico Regionale Sezione Area Sud-Reggio Calabria” (approval number 219, 24 February 2009). All participants gave their informed consent before enrollment.

### 2.1. Study Population

In this analysis, we included a cohort of 1124 hemodialysis (HD) patients who were enrolled from February 2009 to October 2010 as part of the PROGREDIRE (Prospective Registry of The Working Group of Epidemiology of Dialysis Region Calabria) study. This prospective study involved 35 dialysis units across two regions in Southern Italy, specifically Calabria and Sicily. The patients included had been receiving regular HD treatments, with an average frequency of 3 ± 0.4 sessions per week, over a median period of 3.7 years (interquartile range: 1.7 to 7.2 years).

The hemodialysis treatments were conducted using standard bicarbonate dialysis and employed various types of non-cellulosic membrane filters. On average, each dialysis session lasted approximately 231 ± 20 min, with a blood flow rate of 288 ± 36 mL/min. In terms of vascular access, the arteriovenous (AV) fistula was the predominant method, used by 88% of the patients.

Among the study participants, 653 were undergoing treatment with antihypertensive medications. Of these, 310 patients were on monotherapy, using agents such as angiotensin-converting enzyme (ACE) inhibitors, calcium channel blockers, α- and β-blockers, vasodilators, diuretics, or other drugs to manage their blood pressure. Additionally, 209 patients were on dual therapy, 74 on triple therapy, and 60 on a regimen of four or five drugs.

### 2.2. Laboratory Measurements

Blood sampling was performed at baseline after an overnight fast. Blood was drawn during a mid-weekday (brief dialysis interval) in tubes containing EDTA as an anticoagulant. Circulating levels of OPN were centrally measured at the Department of Internal Medicine, University of Genoa, via colorimetric enzyme-linked immunosorbent assay (ELISA), following the manufacturer’s instructions (DuoSet DY1433, R&D Systems, Minneapolis, MN, USA). The lower detection limit was 62.5 pg/mL. To ensure the reliability of our biomarker data and minimize inter-assay and intra-assay variations, we adhered to strict, standardized protocols that controlled for handling variability across all stages, from sample preparation to incubation times, washing steps, and reagent volumes. Additionally, all samples were analyzed within the same assay batch, run in duplicate, and coupled with a full set of calibration standards and controls. Intra- and inter-assay variations were assessed as the coefficient of variation (CV) across duplicate wells for each sample and by comparing the CV across independent assay runs, respectively. We accepted these variations only if they were below 8%.

OPN was assayed from banked (stored at 80°) never-defrost specimens used at baseline, for which plasma was collected in several aliquots to prevent defreezing/refreezing cycles and sample deterioration. Cholesterol, albumin, calcium, phosphate, C-reactive protein (CRP), hemoglobin, alkaline phosphatase, and parathormone (PTH) measurements were made using standard methods in the routine clinical laboratory.

### 2.3. Study Endpoints

The primary endpoints of this study were all-cause mortality and cardiovascular (CV) mortality. Cardiovascular events were centrally adjudicated and classified into several categories. Stroke, which encompassed both ischemic and hemorrhagic types, was confirmed by computed tomography (CT), magnetic resonance imaging (MRI), and/or clinical and neurological evaluations. Transient ischemic attacks (TIAs) were also included in the classification. For myocardial infarctions, diagnoses were confirmed through serial electrocardiogram (ECG) changes and measurements of cardiac biomarkers, providing objective evidence of acute cardiac events. ECG-documented episodes of angina and arrhythmia were similarly recorded. Additionally, sudden unexpected deaths, suspected to be of cardiac origin, were classified under CV mortality. De novo chronic heart failure (CHF) was defined as the development of CHF in patients who were free from this condition at the study baseline. To meet the CHF criteria, patients needed to demonstrate mild or more severe dyspnea during routine activities, corresponding to New York Heart Association (NYHA) class II or higher, along with evidence of left ventricular (LV) disease—either anatomical or functional—confirmed via echocardiography.

All deaths within the study cohort were assessed independently by a panel of three physicians. In cases where the cause of death was uncertain, a consensus-based diagnosis was reached through careful deliberation among the physicians. The adjudication process utilized all available medical information, including hospitalization records, medical charts, and physician notes, to ensure accuracy in classification.

In cases where death occurred outside of a medical facility, interviews with family members or general practitioners were conducted to gather additional context regarding the circumstances leading to the patient’s death. This information helped to clarify potential links to underlying cardiovascular or other health issues, thereby aiding in the accurate classification of the cause of death. This rigorous adjudication process aimed to ensure the reliability of mortality data, which was critical for the study’s assessment of the relationships between clinical characteristics and mortality outcomes in the hemodialysis population.

### 2.4. Statistical Analysis

Data are expressed as mean ± standard deviation (normally distributed data), median and interquartile range (non-normally distributed data), or as percent frequency (categorical data). Pearson’s correlation analysis and linear regression analysis were performed to investigate the independent correlates of OPN. Due to its non-normal distribution, this variable was log-transformed before analysis. Survival analysis was performed by using both univariate and multivariate Cox regression analyses. For multivariate analyses, we adjusted for the full set of traditional risk factors (age, gender, current smoking, diabetes, cholesterol, arterial pressure, antihypertensive treatment, and cardiovascular comorbidities), biomarkers of inflammation and nutritional status [CRP, albumin, body mass index (BMI)], and ESKD-related risk factors (dialysis vintage and hemoglobin). To construct the multivariable Cox regression model, we specifically checked for collinearity among covariates by using the variance inflation factor (VIF), and no multicollinearity was found. As the number of missing values was less than 10% for each variable, in multiple models, we adopted the most conservative approach to replace missing data, i.e., replacement by the mean or median values (according to the data distribution). The date of renal transplantation or last observation for loss-to-follow-up patients was carefully recorded. As these events were not the endpoint of the study, these patients were considered censored in all the models. Effect modification analyses were performed considering the model OPN, the potential effect modifier, and their multiplicative term.

Statistical analysis was performed by using standard statistical packages (SPSS for Windows, Version 28, Chicago, IL, USA) and STATA (Stata Statistical Software: Release 13. College Station, TX, USA: StataCorp LLC).

## 3. Results

The demographic, somatometric, and clinical characteristics of the whole study population are detailed in Table 1. The mean age (SD) was 65 ± 14 years, and 64% of patients were males, 14% were smokers, and 27% were diabetics. Five hundred and eighty-six patients, corresponding to 52%, had previous CV comorbidities. Median OPN levels were 192 ng/mL (IQR 101–329 ng/mL), and the mean calcium and phosphate levels were 9.1 ± 1.0 mg/dL and 5.0 ± 1.6 mg/dL, respectively. Alkaline phosphatase, indicative of bone turnover, had a median level of 88 UI/L (interquartile range: 66–124 UI/L), whereas serum parathyroid hormone (PTH) levels, crucial for understanding bone mineral disorders in ESKD, showed a median value of 243 pg/mL (interquartile range: 118–460 pg/mL).

### 3.1. Independent Correlates of OPN

Pearson’s correlation analysis showed a direct association of OPN with PTH (r = 0.29, *p* < 0.001), alkaline phosphatase (r = 0.22, *p* < 0.001), and phosphate (r = 0.10, *p* = 0.002). In the multivariate linear regression analysis including these correlates, only alkaline phosphatase (β_50units_ = 0.08, *p* < 0.001) and PTH (β_50units_ = 0.03, *p* < 0.001) remained independently associated with OPN (Table 2).

### 3.2. Survival Analysis

During a mean follow-up of 2.8 years, 478 patients died, 271 from CV causes. The univariate analysis revealed that OPN was significantly associated with all-cause mortality (HR 1.15, 95% CI 1.05−1.24, *p* = 0.001) and CV mortality (HR 1.13, 95% CI 1.01−1.26, *p* = 0.03) (Table 3 and Table 4). These associations also held true after adjustment for the full set of traditional risk factors, biomarkers of inflammation and nutritional status, and ESKD-related risk factors (all-cause mortality: HR 1.19, 95% CI 1.09−1.31, *p* < 0.001; CV mortality: HR 1.22, 95% CI 1.08−1.38, *p* = 0.001; Table 3 and Table 4). Further adjustment for PTH and alkaline phosphatase did not affect the strength of the association (all-cause mortality: HR 1.18, 95% CI 1.07−1.30, *p* < 0.001; CV mortality: HR 1.21, 95% CI 1.07−1.37, *p* = 0.003).

**Figure 1:**
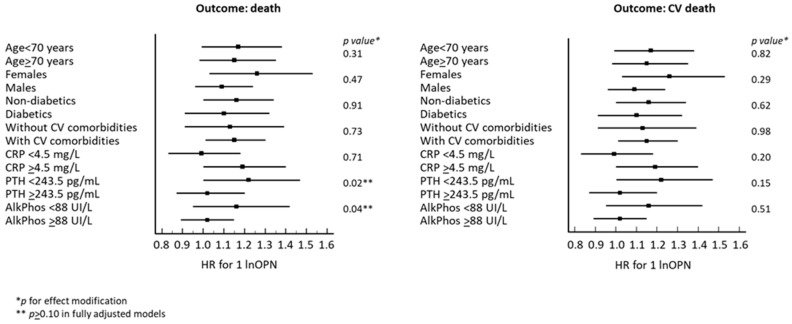
Absence of effect modification on the link between OPN and all-cause (**left** panel) and CV (**right** panel) mortality.

Interaction analysis by age, sex, diabetes, previous CV events, CRP levels, serum PTH, and alkaline phosphatase showed no effect modification on the link between OPN and all-cause/CV mortality (Figure 1).

## 4. Discussion

This study involving a large cohort of 1124 hemodialysis patients demonstrated a significant association between elevated levels of circulating OPN and increased all-cause and CV mortality in patients undergoing hemodialysis. Notably, these associations remained statistically significant even after adjusting for various traditional and ESKD-specific risk factors. These findings suggest that OPN could serve as a valuable biomarker for identifying high-risk patients and guiding therapeutic strategies aimed at improving clinical outcomes.

OPN is involved in numerous biological processes that may contribute to the pathogenesis of cardiovascular diseases in hemodialysis patients. One of its key functions consists of the regulation of mineral metabolism [21] and vascular calcification [22], processes that are frequently dysfunctional in patients with chronic kidney disease. However, the mechanisms by which OPN may promote vascular calcification are multiple. First of all, OPN promotes the differentiation of vascular smooth muscle cells (VSMCs) into an osteoblastic phenotype, leading to calcification. This process is mediated through signaling pathways such as the Wnt/β-catenin pathway, which is activated by OPN [23]. In addition, OPN binds to hydroxyapatite and helps stabilize mineralized matrices, contributing to the deposition of calcium phosphate crystals in vascular tissues [24]. Finally, OPN can inhibit the action of calcification inhibitors like matrix Gla protein (MGP), promoting an environment conducive to vascular calcification [25]. The dysregulation of these processes thus leads to increased vascular stiffness and calcification [26,27,28], both significant risk factors for cardiovascular morbidity and mortality in this population [29]. Furthermore, OPN is known to participate in the inflammatory milieu [30] characteristic of ESKD. As a proinflammatory cytokine, OPN stimulates the production of other inflammatory mediators, such as TNF-α and IL-6 [31]; enhances the recruitment and activation of monocytes and macrophages to sites of injury [32]; and can activate NF-κB [33], a transcription factor that regulates the expression of various inflammatory genes, further perpetuating the inflammatory response. Chronic inflammation has been well documented as a risk factor for cardiovascular diseases [34], and given that OPN is an established inflammatory mediator, it is plausible that this biomarker could play a crucial role in enhancing this risk.

Additionally, OPN has been linked to vascular stiffness [34,35], which increases the workload on the heart and can ultimately lead to heart failure and other adverse cardiovascular events [36]. Vascular stiffness is particularly concerning in hemodialysis patients, as it represents a mechanical alteration in the vascular system that contributes to the overall cardiovascular burden.

As previously reported, previous studies have suggested a role for OPN in vascular calcification and atherosclerosis in the general population [19], but its specific role in hemodialysis patients has not been clearly defined until now. In addition, OPN was found to be associated with inflammation and mortality in a mouse model of polymicrobial sepsis [37]. This association was confirmed in critically hill patients [38] and in patients with septic shock [39] and chronic heart failure [40], but no data are available for hemodialysis patients. This study fills a significant gap in the existing literature by providing compelling evidence that OPN is associated with adverse outcomes in this high-risk population. These findings align with earlier research that identified OPN as a biomarker of inflammation and calcification; however, this study extends those observations specifically to the hemodialysis population.

For instance, a small study conducted by Nitta indicated that OPN is expressed in atherosclerotic lesions and may contribute to vascular calcification in hemodialysis patients [5]. However, this earlier research did not evaluate mortality and cardiovascular events as outcomes, leaving a critical gap in understanding the implications of OPN in this context [37,38,39,40]. While OPN is an established inflammatory mediator [30], consistently associated with atherosclerosis and cardiovascular disease in the general population [19], no cohort studies have previously investigated the link between OPN and all-cause or CV mortality or non-fatal cardiovascular events in hemodialysis patients. Our study is the first to specifically examine this relationship, thereby providing a more comprehensive understanding of the role of OPN in the elevated risk of death and cardiovascular complications observed in this patient group.

In a cohort of more than 1000 HD patients, we found an increased risk of 19% for all-cause and 22% for CV mortality linked to raised OPN levels. In addition, and in spite of the involvement of this biomarker in mineral metabolism, we found that this association also held true after adjustment for PTH and alkaline phosphatase, suggesting a different pathway linking OPN to death. Again, the association between this biomarker and the considered outcome was not modified by the bone status. Vascular calcification is known to have a detrimental effect on cardiovascular events and mortality in dialysis patients [41,42,43,44,45]. Similarly, inflammation has been linked to the same outcomes in several, independent studies [46,47,48]. However, OPN is increasingly recognized as a pleiotropic aging-related molecule, which is difficult to fit into a single pathophysiological pathway [49,50]. Its application as a predictive biomarker indeed ranges from cardiometabolic [51,52,53] to infectious diseases [39,54] and malignancies as well [55,56]. While promising, such a pleiotropic role represents a potential limitation to any targeted therapy.

Despite the significant findings of this study, several limitations warrant consideration. Firstly, the observational nature of the study design means that causality cannot be definitively established between OPN levels and clinical outcomes. Secondly, the data were collected over a specific period and within a limited geographic region, which may limit the generalizability of the results to other populations with different demographics or healthcare systems. Additionally, OPN levels were measured only at baseline, and the absence of temporal variations in OPN levels could influence outcomes.

Furthermore, while adjustments were made for numerous risk factors, the potential for residual confounding remains. Future longitudinal studies incorporating repeated measurements of OPN levels could provide deeper insights into the dynamics of this biomarker and its relationship with clinical outcomes over time. Moreover, interventional studies designed to evaluate whether targeting OPN levels can improve clinical outcomes in hemodialysis patients would be invaluable.

Understanding the specific pathways through which OPN influences cardiovascular risk could also pave the way for the development of new therapeutic strategies aimed at reducing mortality in this high-risk population. In addition, new studies aimed at comparing the impact on mortality of OPN and inflammatory biomarkers, such as CRP and IL-6, could be useful to better understand their different roles and to establish if a multi-biomarker approach would provide better predictive power for clinical outcomes. By elucidating these mechanisms, healthcare providers may be better equipped to identify patients at elevated risk and implement targeted interventions to improve their long-term health outcomes [57].

## 5. Conclusions

In conclusion, this study highlighted that elevated OPN levels are associated with increased all-cause and CV mortality in HD patients. These findings implicate OPN in this population’s high death risk. Mechanistic and intervention studies, such as randomized controlled trials targeting OPN, are needed to confirm these results and explore the hypothesis generated by our study that reducing this biomarker can improve the high death risk of HD patients.

## Figures and Tables

**Table 1:** Main demographic, somatometric, and clinical characteristics of the study population.

	Whole Cohort (*n* = 1124)
Age (years)	65 ± 14
BMI (kg/m^2^)	25 ± 5
Male sex *n* (%)	720 (64)
Current smokers *n* (%)	158 (14)
Past smokers *n* (%)	403 (36)
Diabetics *n* (%)	301 (27)
Antihypertensive treatment *n* (%)	653 (58)
Cardiovascular comorbidities * *n* (%)	586 (52)
Systolic blood pressure (mmHg)	135 ± 22
Diastolic blood pressure (mmHg)	73 ± 12
Dialysis vintage (months)	47 (22–90)
Pre-dialysis potassium (mEq/L)	5.5 ± 0.9
Post-dialysis potassium (mEq/L)	3.9 ± 0.6
Pre-dialysis weight (Kg)	69 ± 15
Post-dialysis weight (Kg)	66 ± 15
Cholesterol (mg/dL)	154 ± 39
HDL cholesterol (mg/dL)	40 ± 13
Hemoglobin (g/dL)	11.3 ± 1.5
Albumin (g/dL)	3.9 ± 0.5
C-reactive protein (mg/L)	4.5 (3.0–12.0)
Calcium (mg/dL)	9.1 ± 1.0
Phosphate (mg/dL)	5.0 ± 1.6
Sodium (mEq/L)	138 ± 4
Alkaline phosphatase (UI/L)	88 (66–124)
PTH (pg/mL)	243 (118–460)

* Cardiovascular comorbidities: the presence, at baseline, of at least one of these comorbidities: angina, arrhythmia, myocardial infarction, coronary surgery, angioplasty, other heart surgery, claudicatio intermittens, amputations, peripheral surgery, stroke, TIAs, and pre-existing chronic heart failure. Data are expressed as mean ± SD, median, and interquartile range, or as percent frequency, as appropriate.

**Table 2:** Multivariate linear regression analysis showing the independent correlates of osteopontin.

Variables	β (95% CI), *p*
PTH (50 pg/mL)	0.03 (0.02, 0.04), *p* < 0.001
Alkaline phosphatase (50 UI/L)	0.08 (0.04, 0.12), *p* < 0.001
Phosphate (mg/dL)	0.03 (−0.01, 0.07), *p* = 0.16

**Table 3:** Crude and adjusted hazard rate (HR) of all-cause mortality for osteopontin.

Variables.	HR (95% CI), *p*	HR (95% CI), *p*
ln osteopontin (1 ng/mL)	1.15 (1.05–1.24), *p* = 0.001	1.19 (1.09–1.31), *p* < 0.001
Age (1 year)		1.05 (1.04–1.05), *p* < 0.001
Gender (0 = female, 1 = male)		1.13 (0.93–1.38), *p* = 0.21
Current smoking (0 = no, 1 = yes)		0.98 (0.73–1.31), *p* = 0.88
Diabetes (0 = no, 1 = yes)		1.31 (1.06–1.61), *p* = 0.01
Cholesterol (1 mg/dL)		1.00 (0.99–1.00), *p* = 0.003
Pre-dialysis systolic blood pressure (1 mm/Hg)		1.00 (1.00–1.00), *p* = 0.90
Previous CV comorbidities (0 = no, 1 = yes)		1.45 (1.20–1.77), *p* < 0.001
Dialysis vintage (1 day)		1.00 (1.00–1.00), *p* < 0.001
Blood pressure lowering therapy (0 = no, 1 = yes)		1.02 (0.84–1.25), *p* = 0.81
Albumin (1 g/dL)		0.77 (0.63–0.94), *p* = 0.01
Calcium (1 mg/dL)		1.04 (0.94–1.15), *p* = 0.44
Phosphate (1 mg/dL)		0.99 (0.93–1.05), *p* = 0.73
Hemoglobin (1 g/dL)		0.95 (0.89–1.02), *p* = 0.15
C-reactive protein (1 mg/L)		1.00 (1.00–1.002), *p* = 0.84
Body mass index (1 Kg/m^2^)		0.97 (0.97–1.02), *p* = 0.77

**Table 4:** Crude and adjusted hazard rate (HR) of all-cause mortality for osteopontin.

Variables	HR (95%CI), *p*	HR (95%CI), *p*
ln osteopontin (1 ng/mL)	1.13 (1.01–1.26), *p* = 0.03	1.22 (1.08–1.38), *p* < 0.001
Age (1 year)		1.04 (1.03–1.06), *p* < 0.001
Gender (0 = female, 1 = male)		1.30 (1.00–1.70), *p* = 0.06
Current smoking (0 = no, 1 = yes)		0.81 (0.54–1.23), *p* = 0.25
Diabetes (0 = no, 1 = yes)		1.49 (1.14–1.95), *p* = 0.01
Cholesterol (1 mg/dL)		1.00 (0.99–1.00), *p* = 0.002
Pre-dialysis systolic blood pressure (1 mm/Hg)		1.00 (0.99–1.01), *p* = 0.99
Previous CV comorbidities (0 = no, 1 = yes)		1.71 (1.31–2.24), *p* < 0.001
Dialysis vintage (1 day)		1.00 (1.00–1.00), *p* = 0.09
Blood pressure lowering therapy (0 = no, 1 = yes)		1.06 (0.82–1.38), *p* = 0.82
Albumin (1 g/dL)		0.88 (0.68–1.14), *p* = 0.44
Calcium (1 mg/dL)		1.00 (0.87–1.15), *p* = 0.99
Phosphate (1 mg/dL)		0.96 (0.88–1.04), *p* = 0.34
Hemoglobin (1 g/dL)		0.97 (0.89–1.05), *p* = 0.38
C-reactive protein (1 mg/L)		1.00 (1.00–1.003), *p* = 0.97
Body mass index (1 Kg/m^2^)		1.00 (0.96–1.03), *p* = 0.81

## Data Availability

The dataset presented in this article is not readily available because includes clinical data. Reasonable requests to access the datasets should be directed to the corresponding author.
